# Spoof surface plasmon polaritons in terahertz transmission through subwavelength hole arrays analyzed by coupled oscillator model

**DOI:** 10.1038/srep16440

**Published:** 2015-11-09

**Authors:** Shan Yin, Xinchao Lu, Ningning Xu, Shuang Wang, Yiwen E., Xuecong Pan, Xinlong Xu, Hongyao Liu, Lu Chen, Weili Zhang, Li Wang

**Affiliations:** 1 Beijing National Laboratory for Condensed Matter Physics, Institute of Physics, Chinese Academy of Sciences, Beijing 100190, China; 2 Key Laboratory of Microelectronics Devices & Integrated Technology, Institute of Microelectronics of Chinese Academy of Sciences, Beijing 100029, China; 3 School of Electrical and Computer Engineering, Oklahoma State University, Stillwater, Oklahoma 74078, USA; 4 School of Electronic Engineering, Tianjin University of Technology and Education, Tianjin 300222, China; 5 State Key Lab Incubation Base of Photoelectric Technology and Functional Materials, Institute of Photonics & Photon-Technology, Northwest University, Xi’an 710069, China

## Abstract

Both the localized resonance and excitation of spoof surface plasmon polaritons are observed in the terahertz transmission spectra of periodic subwavelength hole arrays. Analyzing with the coupled oscillator model, we find that the terahertz transmission is actually facilitated by three successive processes: the incident terahertz field first initiates the localized oscillation around each hole, and then the spoof surface plasmon polaritons are excited by the localized resonance, and finally the two resonances couple and contribute to the transmission. Tailoring the localized resonance by hole size, the coupling strength between spoof surface plasmon polaritons and localized resonances is quantitatively extracted. The hole size dependent transmittance and the coupling mechanism are further confirmed by fitting the measured spectra to a modified multi-order Fano model.

Plasmonic devices fabricated in metal-based surface convert the energy from photons to oscillating electrons, e.g. Surface Plasmon Polaritons (SPPs)^1^,^2^,^3^,^4^. As SPPs can be excited in artificial materials and tailored by geometric configurations, plasmonic devices are flexibly integrated into an instrument on-chip that is versatile, miniature, and easy to be manufactured. At terahertz (THz) frequencies, although SPPs are weakly bounded to the metal-dielectric interface due to the fact that metals behave as a near perfect electric conductor (PEC), spoof SPPs can be achieved by introducing surface configurations. Pendry and Hibbins *et al.* have demonstrated that the dispersion of the spoof SPPs could be tailored by both hole size and periodicity on PECs^5^,^6^. Two different resonances, i.e., localized resonance depending on the hole size and spoof SPP (abbreviates to SPP hereinafter) resonance arising from the surface periodicity, were considered as contributors to the THz transmission through periodic arrays of subwavelength holes^7^. THz transmission in random subwavelength hole arrays was also investigated and the resonance frequencies determined only by the hole sizes was observed^8^. By changing the hole length, the dominant resonant transmission mechanism from localized resonance to excitation of surface waves and vice versa was tuned^9^. Though the localized resonance and SPP resonance are deemed to be the mechanisms leading to THz transmission through subwavelength hole arrays, their mode nature and quantitative analysis to their relation still remain unexplored.

Characterized by asymmetric line shapes, which is configured by the constructive and destructive interactions between broad continuum state and narrow discrete resonances^10^,^11^,^12^, Fano interferences are commonly found in the ionization of atoms^13^, quantum transports^14^ and plasmonic nanostructures^15^. THz transmission through subwavelength hole arrays also shows asymmetric resonances with Fano profile, which was explained by the interaction between the continuum nonresonant transmission and SPP resonance^16^ while the contribution from localized resonance was ignored. Lacking of further information on the detailed physical processes behind the THz transmission, especially the excitation mechanism of each mode, the analysis with Fano model is restricted. Alternatively, the coupled oscillator model provides an intuitive and quantitative method to investigate the original nature of Fano coupling between the localized resonance and SPP resonance^17^,^18^, and also their interactions with the external electromagnetic waves, which opens a door for the in-depth exploration on the THz transmission through subwavelength hole arrays.

In this study, THz transmission through random and periodic rectangular hole arrays are measured to discriminate and attribute the contributions from localized resonance and SPP resonance, respectively. Based on the analysis of the coupled oscillator model, the physical mechanism behind the THz transmission through subwavelength periodic hole arrays is fundamentally revealed: the localized resonance acts as the dominant role for harvesting the incident THz waves, then excites the SPP resonance and finally the two resonances couple to facilitate the THz transmission. The quantitative analysis manifests that the strong coupling between SPP and localized resonances can be realized by tailoring the hole size to align the two resonances, which is essentially caused by the variation of the localized resonance for different hole sizes. Also, the hole size dependent asymmetry of the Fano coupling is characterized by the modified multi-order Fano model. The consistent quantitative analysis derived from the two models verifies the excitation of the resonance modes and coupling mechanism of the THz transmission through periodic subwavelength hole arrays. The application of the coupled oscillator model in THz domain also can be utilized in other frequency regimes.

## Results and Discussion

**Figure 1:**
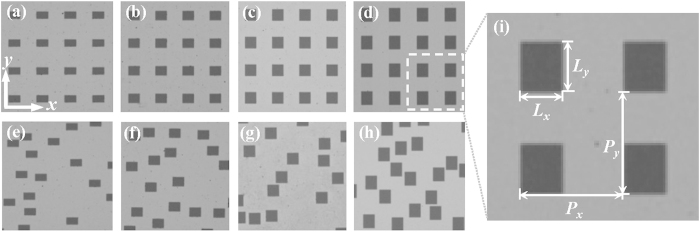
Microscopic images of the periodic hole arrays with *P*_*x*_ = *P*_*y*_ = 240 *μ*m, *L*_*x*_=100 *μ*m, *L*_*y*_ = 60 *μ*m (**a**), 80 *μ*m (**b**), 100 *μ*m (**c**) and 120 *μ*m (**d**), and the corresponding random hole arrays with the same hole size and filling factors (**e–h**).

The random and periodic rectangular hole arrays for our experimental studies are illustrated in Fig. 1. The subwavelength hole arrays are perforated in an aluminum film deposited on a 640 *μ*m-thick silicon substrate. The thickness of the aluminum film is 200 nm which is much thicker than the THz skin depth. The length in the *x* direction of the holes (*L*_*x*_) fixes at 100 *μ*m, while the length in the *y* direction (*L*_*y*_) varies from 60 *μ*m to 120 *μ*m at an interval of 20 *μ*m. For periodic hole arrays, the *x* and *y* direction have the same periodicity *P* of 240 *μ*m. The corresponding random hole arrays hold an effective period (*EP*) equaling to *P*, which maintains the same hole sizes and filling factors as the periodic samples.

**Figure 2:**
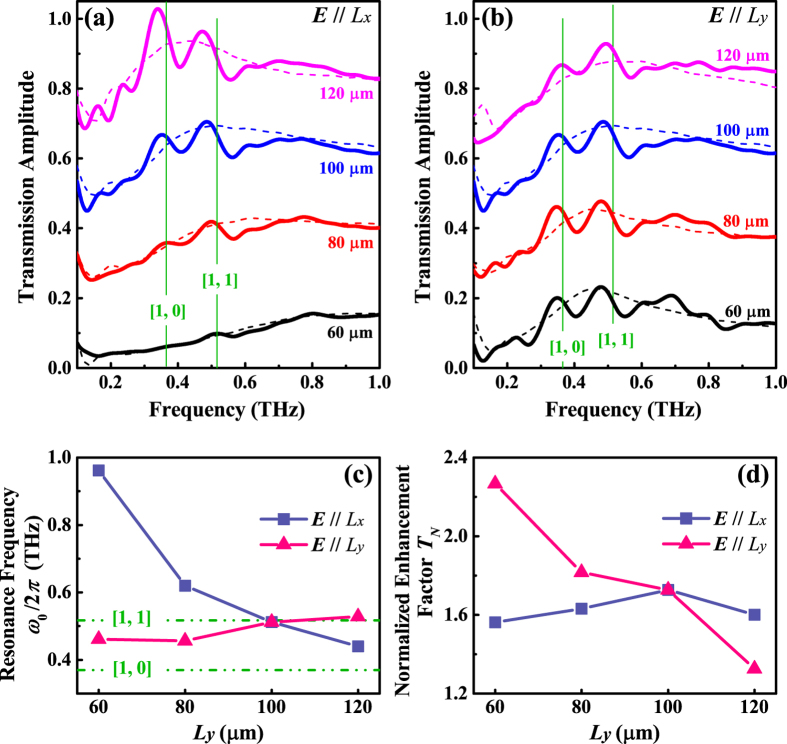
Measured transmission spectra of the periodic (solid lines) and random (dash lines) hole arrays with *L*_*x*_ = 100 *μ*m and different *L*_*y*_ for ***E***//*L*_*x*_ (**a**) and ***E***//*L*_*y*_ (**b**). For clarification, the transmission spectra with different *L*_*y*_ are vertically shifted by 0.2 (80 *μ*m), 0.4 (100 *μ*m) and 0.6 (120 *μ*m). The localized resonance frequency *ω*_0_ (**c**) and normalized enhancement factor *T*_*N*_(**d**) obtained from the measured spectra of the random hole arrays with different *L*_*y*_ and hole orientations. The theoretical values of the SPP resonance frequencies of the [0, 1] and [1, 1] modes are indicated in (**a**,**b**) with vertical solid lines, and in (**c**) with horizontal dashed lines.

A standard THz time-domain spectroscopy (THz-TDS) system^19^ is used to measure the transmission spectra at normal incidence under nitrogen environment. The THz radiation is generated by a photoconductive antenna driven by ultrafast laser pulses (70 fs duration, 800 nm central wavelength and 80 MHz repetition rate) and detected by a <110> ZnTe crystal. The transmission spectra of the random hole arrays (dash lines) of varying *L*_*y*_ with different hole orientations, i.e. ***E***//*L*_*x*_ and ***E***//*L*_*y*_, are displayed in Fig. 2(a,b), respectively. The broad resonances of the random hole arrays can be regarded as the multiple individual hole resonances, i.e., localized resonance related to the hole shape^8^, which basically reflects the behavior of the localized charge oscillation around a single hole. The central frequency *ω*_0_ of the localized resonance and the normalized enhancement factor *T*_*N*_ obtained by dividing the maximum transmission by the filling factor *FF* = *L*_*x*_*L*_*y*_/*P*^2^ are illustrated in Fig. 2(c,d). As the size *L*_*y*_increases, for ***E***//*L*_*x*_, *ω*_0_ shows significant red-shift but *T*_*N*_ remains almost unchanged; for ***E***//*L*_*y*_, however, *ω*_0_ does not shift too much but *T*_*N*_ proportionally decreases (the fluctuation in the data may come from the limited numbers of random holes covered by the incident THz beam with a moderate diameter of 4 mm). The dependence of *ω*_0_ and *T*_*N*_ on hole sizes agrees well with previous reports in refs 8, 20, 21: *ω*_0_ is determined by the hole length perpendicular to the ***E*****-**field, and *T*_*N*_ is dependent on the hole length parallel to the ***E*****-**field. The spectra of the periodic hole arrays overlapping with the corresponding spectra of the random samples are also shown in Fig. 2(a,b). Regardless of hole orientations, several narrow resonances superimposing on the broad spectra of random arrays indicate the feature of multi order SPP modes. The theoretical values of SPP resonance frequencies for the [0, 1] and [1, 1] modes, which are marked by the vertical lines, are always higher than the measured peak frequencies. The sharp resonant peaks also show a stronge asymmetry in the vicinity of *ω*_0_. All these observations manifest the Fano coupling between the SPP resonance and broad localized resonance^22^.

### The coupled oscillator model

**Figure 3:**
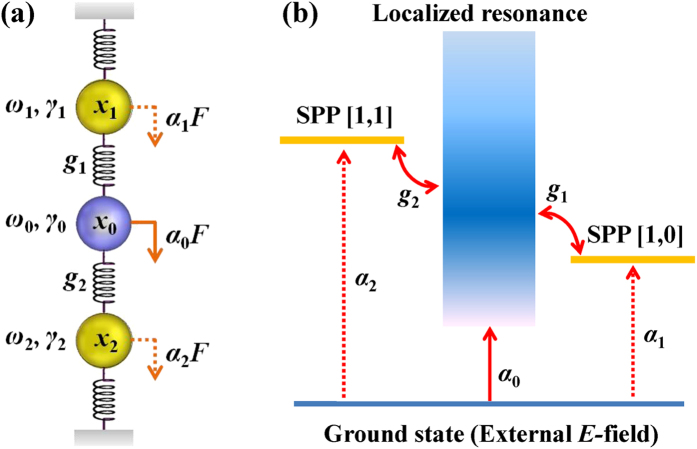
(**a**) Schematic of coupled oscillator model. The oscillators correspond to the localized resonance (*x*_0_), SPP [0, 1] (*x*_1_) and SPP [1, 1] (*x*_2_) modes. (**b**) Fano process with a localized resonance (continuum state), SPP [0, 1] and [1, 1] modes (discrete states), excited by the incident THz field. *ω*_0_, *ω*_1_ and *ω*_2_ are the resonance frequencies, *γ*_0_, *γ*_1_ and *γ*_2_ are the damping rates, *α*_0_, *α*_1_ and *α*_2_ represent the excitation efficiencies, *F* is the external field, *g*_1_ and *g*_2_ are the coupling coefficients.

To reveal further details about the excitation of different modes and the interaction among them, a coupled oscillator model is set up (see Fig. 3(a)). For simplicity, it is assumed that only two SPP modes (with resonance frequency *ω*_1_, *ω*_2_) closest to the localized resonance frequency (*ω*_0_) are taken into account, and no coupling occurs between the SPP modes. The localized resonance mode, SPP [0, 1] and [1, 1] modes are all regarded as classical oscillators and denoted by 0, 1 and 2, respectively. The coupling between the SPP modes and localized resonance is denoted by the coupling coefficients *g*_1_ and *g*_2_. It is supposed that all the three modes could be excited by accepting energy from the incident THz field directly, and the efficiency is described by a coefficient *α*_*n*_(*n* = 0, 1, 2). Therefore, the equations of motion can be expressed as follows,


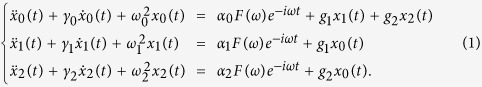


In the equations, *x*_*n*_represents the amplitude of the charge oscillation for each mode, which takes harmonic form as 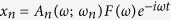, and *A*(*ω*) is the complex amplitude in the frequency domain. *F*(*ω*)*e*^*-iωt*^ represents the external field exerting on the oscillators time-harmonically. *γ*_*n*_stands for the damping rate of each mode. A group of special solutions of the Eq. (1) is obtained straightforward as follows,


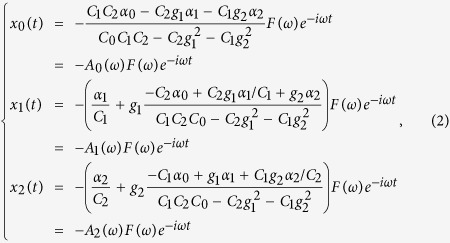


where 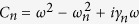. For the spectral amplitudes *A*_1_(*ω*) and *A*_2_(*ω*), the first term (in brackets) produces a Lorentzian oscillation strongly associated with *α*_1_ and *α*_2_, and represents the probability excited directly by the incident field; the second term describes the Fano resonance resulted from the coupling between the SPP modes and localized resonance mode. The THz transmission through subwavelength holes is attributed to the reemission of the resonance modes, whose radiating efficiency is proportional to the second derivative of the replacement of the charge 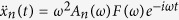, and the frequency domain counterparts can be expressed by 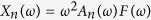. *F*(*ω*) can be regarded as the frequency spectrum of reference signal measured in THz-TDS experiments. After dividing expressions of *X*_*n*_(*ω*) from Eq. (2) by *F*(*ω*), like the ordinary treatment made in the THz-TDS analysis, the measured transmission spectra can be fitted by the following equation,


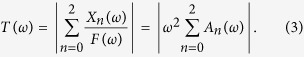


In fitting processes, the SPP resonance frequency for each mode is taken from the theoretical calculation: *ω*_1_/2*π* = 0.37 THz, *ω*_2_/2*π* = 0.517 THz; initial values of *γ*_0_, *ω*_0_ and *α*_0_ (listed in supplementary material) are obtained independently by fitting the measured transmission spectra of random hole arrays, while *A*_1_(*ω*) and *A*_2_(*ω*) in Eq. (3) are set to be zero. To get better fitted results, both the values of *γ*_1_ and *γ*_2_ are set to be 0.067 ± 0.003 THz.

**Figure 4:**
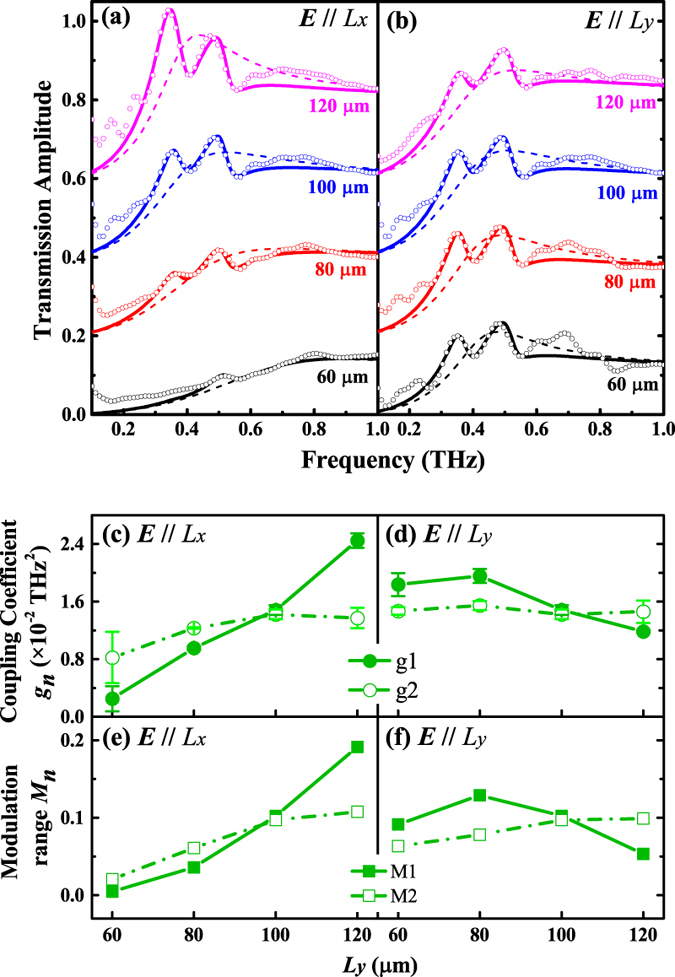
Fitted (solid lines) and measured (open dots) transmission spectra of the periodic hole arrays superimpose the fitted transmission spectra of the random hole arrays (dashed lines) with different *L*_*y*_ for *E*//*L*_*x*_ (**a**) and *E*//*L*_*y*_ (**b**). For clarification, the transmission spectra with different *L*_*y*_ are vertically shifted by 0.2 (80 *μ*m), 0.4 (100 *μ*m) and 0.6 (120 *μ*m). Extracted coupling coefficient *g*_1_, *g*_2_ (**c**) and measured transmission modulation *M*_1_, *M*_2_ (**e**) dependent on hole size for ***E***//*L*_*x*_; *g*_1_, *g*_2_ (**d**) and *M*_1_, *M*_2_ (**f**) for ***E***//*L*_*y*_.

The transmission spectra described by Eq. (3) using fitted coefficients are shown in Fig. 4(a,b), which agree well with the measurements in the vicinity of the resonant frequencies. With the fitted transmission spectra, we first realize the quantitative analysis of the efficiency of each mode excited by the external ***E*****-**field. The fitted values of *α*_1_ and *α*_2_ are listed in Table 1 (the fitted values of other parameters are listed in Supplementary Table SI online). For all fitted results from different samples, *α*_1_ and *α*_2_ are at least two orders of magnitude smaller than *α*_0_. It tells that the efficiency of the SPP modes directly excited by the external ***E*****-**field is much less than that of the localized resonance mode. In fact, the direct excitation of the SPP modes by incident THz waves is virtually prohibited. To further check this issue, we set *α*_1_ = *α*_2_ = 0, and the transmission is simplified as


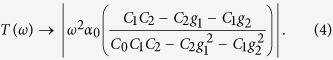


Comparing to the results using fitted finite values of *α*_1_ and *α*_2_, we find that the change of fitted transmission spectra described by Eq. (4) is almost indistinguishable (see Supplementary Fig. S1 online), which verifies that the SPP modes are not excited by the external ***E*****-**field directly. Instead, SPPs have to be excited by the localized resonance through energy transfer process. We also analyze the transmission with varying *α*_1_, *α*_2_ values comparable to that of *α*_0_, and the impact weight of the SPP modes gets stronger with increasing *α*_1_ and *α*_2_, which manifests the situation in the optical transmission through subwavelength hole arrays (see Supplementary Fig. S2 online).

After clarifying the mechanism of THz transmission through subwavelength hole arrays, we can find more evidence via quantitative analyzing the coupling between the SPP resonance and localized resonance. Since the SPP mode is excited from the localized resonance mode, it is natural that when the two modes get closer, more energy of the localized resonance mode can be transferred to the SPP mode, and more likely the coupling will occur. Therefore, the change of the localized resonance with different hole sizes should essentially lead to the varying feature of the coupling in the periodic hole arrays. To verify our inference, we first extract the coupling coefficient *g*_*n*_(*n* = 1, 2 for *g*_*n*_) from the fitted transmission spectra, which is shown in Fig. 4(c,d) with error bar. Here *g*_*n*_ is taken as the mean value of different fittings with varying initial values, including the fittings with Eqs (3) and (4), and the error bar indicates the standard deviation. Except the scattered values of *g*_1_ for *L*_*y*_ = 60 *μ*m and ***E***//*L*_*x*_, due to the weak coupling, the fluctuation of *g*_*n*_ is quite small, which means our fittings are reliable and robust, and the simplification of Eq. (4) is also verified. With increasing *L*_*y*_, *g*_1_ and *g*_2_ both increase monotonically for ***E***//*L*_*x*_ but keep less changed for ***E***//*L*_*y*_. Associated with the spectra of the localized resonance, it is obvious that the variation of the coupling strength is greatly influenced by the variation of the localized resonance. This fact coincides with the coupling mechanism. For ***E***//*L*_*x*_, as *L*_*y*_ increases, the localized resonance frequency *ω*_0_ undergoes red-shift and approaches resonant frequencies of the SPP modes, which results in the stronger excitation of the SPP resonance and then the stronger coupling between the SPP and localized resonance modes. Also, since the SPP [0, 1] mode holds the same resonant direction with E-field of localized resonance mode, the coupling strength of the SPP [0, 1] mode is stronger than SPP [1, 1] mode intrinsically. Accordingly, *g*_1_ exceeds *g*_2_ with *L*_*y*_ = 120 *μ*m, ***E***//*L*_*x*_, as the localized resonance frequency *ω*_0_ sits between *ω*_1_ and *ω*_2_. On the other hand, the modulation of the transmission enhancement and suppression, which induced by constructive and destructive interference between SPP modes and localized resonance, also correlates to the coupling strength. We measure the modulation range *M*_*n*_ (the difference between the maximum and minimum values, and *n* = 1, 2 for *M*_*n*_) around each SPP mode from the transmission difference spectrum of the periodic and random hole arrays (See Fig. S3). As shown in Fig. 4(e,f), the variation of *M*_*n*_ dependent on hole size is consistent with that of the coupling coefficient *g*_*n*_, which is another support verifying the mechanism we mentioned. Now it is confirmed that the coupling strength is mainly affected by the localized resonance frequency shift tuned by the hole size. By optimizing the hole size, the SPP and localized resonance modes can be coincided to produce stronger coupling.

### The Fano model

As the Fano coupling plays a pivotal role in the THz transmission, to reconfirm our conclusions initiated from the coupled oscillator model, we analyze the THz transmittance through subwavelength hole arrays with the Fano model^10^. The narrow SPP modes and the broad localized resonance mode act as discrete states and continuum state, respectively (see Fig. 3(b)). As the Fano model describes the energy relation, the modified multi-order Fano model as follows has been applied to fit the transmittance spectra of the periodic hole arrays (see the details in the Method section):


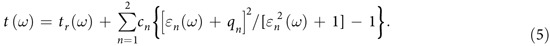


where *t*_*r*_is the background transmittance from the random hole arrays, 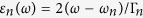 (*n* = 1, 2 in Fano model), *ω*_*n*_ and Γ_*n*_ denote the resonance frequency and linewidth for each SPP mode, respectively. The weighting factor *c*_*n*_ represents the contribution to the THz transmission from each coupled SPP mode. The asymmetry parameter *q*_*n*_ represents the ratio of the transition probabilities to the SPP resonance and localized resonance. The asymmetry of spectra around the SPP modes is determined by *q*_*n*_ and *c*_*n*_ together: the former depicts the line shape and the latter adjusts the magnitude.

**Figure 5:**
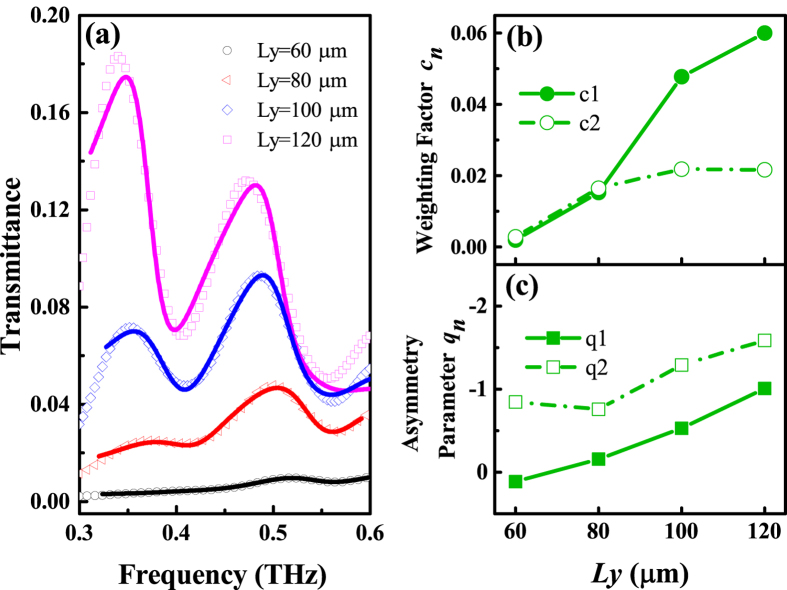
Fitted (solid lines) and measured (open dots) transmittance spectra of periodic hole arrays with different *L*_*y*_ for ***E***//*L*_*x*_(**a**). Extracted weighting factor *c*_*n*_ (**b**) and asymmetry parameter *q*_*n*_ (**c**) from the fitted results.

The experimental transmittance spectra of the periodic hole arrays in the vicinity of SPP resonance frequencies of the [0, 1] and [1, 1] modes are fitted by Eq. (5). Since the spectral features of different hole sizes under ***E***//*L*_*y*_ are similar, we focus on the characters of the transmittance under ***E*****//***L*_*x*_. As shown in Fig. 5(a), the Fano model fitted results (solid lines) agree well with the measurements data (open dots), and the spectrum asymmetry around SPP modes shows more and more distinct with increasing *L*_*y*_. The *c*_*n*_ extracted from the Fano model fitted results is shown in Fig. 5(b). As *L*_*y*_ increases, *c*_1_ increases monotonically while *c*_2_ increases slowly and even stops down when *L*_*y*_ reaches 120 *μ*m. The increasing *c*_*n*_ manifests that more coupled SPP mode contributes to the THz transmission with increasing *L*_*y*_, which coincides with the change rules of coupling coefficient *g*_*n*_ as we concluded. In addition, the asymmetry parameter |*q|* in Fano model are extracted. As shown in Fig. 5(c), as *L*_*y*_ increases, both |*q*_1_| and |*q*_2_| increase, which means that the ratio of the transition probabilities to the SPP resonance and localized resonance rises. Note that due to the non-uniform background from the localized resonance in our complex system, it is unlikely to obtain the absolute transition probability to the SPP resonance. Joint with the Fano model, the availability of the coupled oscillator model and the conclusions we discussed are evidenced.

In summary, by comparing the THz transmission through random and periodic rectangular hole arrays, the contributions from localized resonance and SPP resonance are discriminated. With the coupled oscillator model, the physical mechanism of SPPs in THz transmission is revealed that the SPP resonance is excited by the localized resonance, and induces the THz transmission via coupling with the localized resonance. With quantitatively analysis, the stronger coupling can be achieved by tailoring the hole size to coincide the SPP and localized resonance modes. The applicability of the coupled oscillator model is also verified by the multi-order Fano model. This study will provide a theoretical guidance to the SPPs tailored by subwavelength structures and pave the way to developing plasmonic devices, for instance, sensors, absorbers and filters in the THz regime. Moreover, the analytical methods can also be extended to study the transmission enhancement in other wavelength regions.

## Methods

### Modified multi-order Fano model

As shown in ref. [14], the typical Fano curve can be represented by the function *f*(*q*; *ε*) as follows,


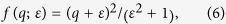


where 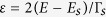, *E*_*s*_and Γ_*s*_ represent the energy and the spectra width of the discrete state, respectively; dimensionless *q* is the asymmetry parameter; |*q*| indicates the ratio of the transition probabilities to the discrete state and to the continuum state. Notice that the baseline of the Fano curve is normalized—it approaches 1 in the region away from the resonance center of the discrete state—if Γ_*s*_ is less than *E* and the values of |*q*| is not too large.

In our system, the discrete states, i.e. the SPP modes, are excited by the continuum state, i.e. the localized resonance mode. Since the Fano model describes the energy relation, the transmittance spectra are utilized in the fitting. By substituting 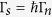 in Eq. (6), with the assistant background transmittance *t*_*r*_ from the random hole arrays, the transmittance of the periodic hole arrays can be expressed by the multi-order Fano model as follows:


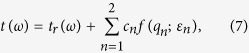


where 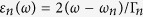, *ω*_*n*_ is the resonance frequency, Γ_*n*_ is the linewidth, *q*_*n*_ is the asymmetry parameter and *c*_*n*_ is the weighting factor for each SPP mode. Here, *c*_*n*_ will not only modify the magnitude of *f*(*q*_*n*_; *ε*_*n*_), but also shift the baseline from 1 to *c*_*n*_. As a result, the term of the sum in Eq. (7) will shift the baseline away from *t*_*r*_(*ω*) with an additional value of 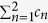, which is unexpected and needs to be corrected. By subtracting the additional value, we finally derive the modified multi-order Fano model [Eq. (5) in the main body] as follows:


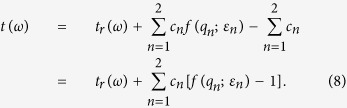


This model is also valid for the situation that more than two discrete states couple to a continuum state, as long as the background profile from the continuum state is provided.

## Additional Information

**How to cite this article**: Yin, S. *et al.* Spoof surface plasmon polaritons in terahertz transmission through subwavelength hole arrays analyzed by coupled oscillator model. *Sci. Rep.* **5**, 16440; doi: 10.1038/srep16440 (2015).

## Supplementary Material

Supplementary Information

## Figures and Tables

**Table 1:** Extracted parameter *α*_*n*_ (*n* = 0, 1, 2) from the fitted transmission spectra in Fig. 4(a,b).

		*α*_0_	*α*_1_	*α*_2_
***E*****//***L*_*x*_	60 *μ*m	0.11	2.7e-3	3.7e-3
	80 *μ*m	0.193	3.1e-3	7.3e-4
	100 *μ*m	0.192	3.0e-3	2.5e-4
	120 *μ*m	0.201	1.6e-3	2.2e-14
***E*****//***L*_*y*_	60 *μ*m	0.118	5.0e-14	2.0e-14
	80 *μ*m	0.161	2.0e-3	3.3e-7
	100 *μ*m	0.192	3.0e-3	2.5e-4
	120 *μ*m	0.208	5.5e-3	4.2e-3
