# Supplementary material for: Spoof surface plasmon polaritons in terahertz transmission through subwavelength hole arrays analyzed by coupled oscillator model
Source: Sci Rep. 2015 Nov 9;5:16440. doi: 10.1038/srep16440 (PMC4637906; doi:10.1038/srep16440)
Supplement: Supplementary Information [file srep16440-s1.pdf]

Supplementary information

Spoof surface plasmon polaritons in terahertz transmission through subwavelength hole arrays analyzed by coupled oscillator model

Shan Yin[1], Xinchao Lu[2,*], Ningning Xu[3], Shuang Wang[4], Yiwen E[1], Xuecong Pan[1], Xinlong Xu[5], Hongyao Liu[2], Lu Chen[2], Weili Zhang[3], and Li Wang[1]

[1] Beijing National Laboratory for Condensed Matter Physics, Institute of Physics, Chinese Academy of Sciences, Beijing 100190, China

[2] Key Laboratory of Microelectronics Devices & Integrated Technology, Institute of Microelectronics of Chinese Academy of Sciences, Beijing 100029, China

[3] School of Electrical and Computer Engineering, Oklahoma State University, Stillwater, Oklahoma 74078, USA

[4] School of Electronic Engineering, Tianjin University of Technology and Education, Tianjin 300222, China

[5] State Key Lab Incubation Base of Photoelectric Technology and Functional Materials, Institute of Photonics & Photon-Technology, Northwest University, Xi'an 710069, China

*Email address: luxinchao@ime.ac.cn

Coupled oscillator model fitted results

In this section, we present more details about the fitted results from the coupled oscillator model. As shown in Figs. S1(a) and S1(b), regardless of hole orientations, the fitted transmission spectra with non-zero α_1, α_2 (solid lines) and zero α_1, α_2 (open dots) with Eq. (3) are quite similar and most identical, and the fitted results with non-zero α_1, α_2 are plotted in Figs. 3(a) and 3(b) in the main body. All parameters extracted from the fitted transmission spectra are listed in Table SI and SII. Though α_1 and α_2 are set to be zero, there is no essential difference for all parameters in the two groups of fitted results. That means the spoof surface plasmon polariton (SPP) resonances excited by the external $\boldsymbol{E}$-field is insignificant for the THz transmission in our system.

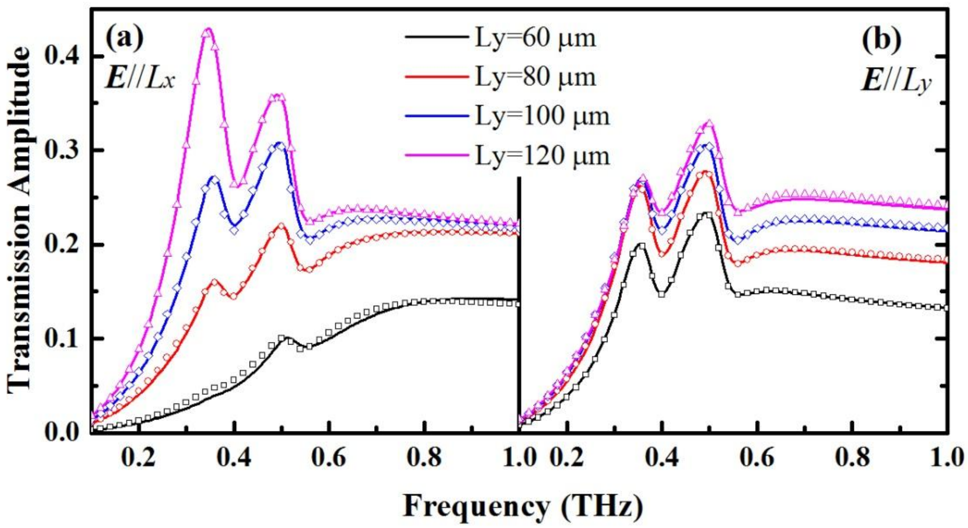

Fig. S1. Fitted transmission spectra with non-zero α_1, α_2 (solid lines) and zero α_1, α_2 (open dots) with Eq. (3) for configuration $\boldsymbol{E}//L_x$ (a) and $\boldsymbol{E}//L_y$ (b).

TABLE SI. Extracted parameters from the fitted transmission spectra in Fig. S1 for varying L_y with non-zero α_1, α_2.

		α_1	α_2	α_0	γ_0 (THz)	$\omega_0/2\pi$ (THz)	γ_1 (THz)	γ_2 (THz)	g_1 (THz^2)	g_2 (THz^2)
$\boldsymbol{E}//L_x$	60 μm	2.7e-3	3.7e-3	0.11	0.610	0.700	0.067	0.070	0.0010	0.0040
	80 μm	3.1e-3	7.3e-4	0.193	0.500	0.496	0.067	0.065	0.0092	0.0123
	100 μm	3.0e-3	2.5e-4	0.192	0.330	0.430	0.067	0.065	0.0146	0.0148
	120 μm	1.6e-3	2.2e-14	0.201	0.230	0.400	0.067	0.065	0.0260	0.0140
$\boldsymbol{E}//L_y$	60 μm	5.0e-14	2.0e-14	0.118	0.249	0.439	0.067	0.067	0.0195	0.0152
	80 μm	2.0e-3	3.3e-7	0.161	0.287	0.430	0.067	0.067	0.0193	0.0152
	100 μm	3.0e-3	2.5e-4	0.192	0.330	0.430	0.067	0.065	0.0146	0.0148
	120 μm	5.5e-3	4.2e-3	0.208	0.360	0.435	0.070	0.067	0.01183	0.0155

TABLE SII. Extracted parameters from the fitted transmission spectra in Fig. S1 for varying L_y with zero α_1, α_2.

		α_1	α_2	α_0	γ_0 (THz)	$\omega_0/2\pi$ (THz)	γ_1 (THz)	γ_2 (THz)	g_1 (THz^2)	g_2 (THz^2)
$\boldsymbol{E}//L_x$	60 μm	0	0	0.112	0.550	0.640	0.067	0.070	0.0040	0.0115
	80 μm	0	0	0.201	0.500	0.473	0.067	0.065	0.0095	0.0112
	100 μm	0	0	0.192	0.330	0.420	0.067	0.065	0.0143	0.0144
	120 μm	0	0	0.204	0.230	0.400	0.067	0.065	0.0260	0.0140
$\boldsymbol{E}//L_y$	60 μm	0	0	0.117	0.245	0.44	0.067	0.065	0.0200	0.0146
	80 μm	0	0	0.167	0.293	0.423	0.067	0.067	0.0187	0.0152
	100 μm	0	0	0.200	0.330	0.420	0.067	0.065	0.0143	0.0144
	120 μm	0	0	0.220	0.342	0.430	0.070	0.067	0.0121	0.0128

Excitation mechanism of SPPs

As we discussed in the main body, α_n (n = 0, 1, 2) represents the probable excitation directly by the incident field. To further explore the excitation mechanism of SPPs, we calculate the transmission spectra with varying values of α_1 and α_2 using Eq. (3). The normalized transmission spectra are shown in Fig. S2, where $\alpha_1 = \alpha_2$ is set for simplification. It is found that, as the values of α_1 and α_2 increase, the asymmetry of the line shape around the SPP modes reduces gradually, namely, the spectra change from the Fano line shape to Lorentzian shape and the resonant peak positions approach the theoretical values of the SPP modes, which reflects the dominant excitation of the SPP resonance transforms from the localized resonance to the external field. From Eq. (2), this result can be understood as the change in the relative weight of the two terms in the solutions of $A_1(\omega)$ and $A_2(\omega)$. As the increase of the SPPs excited by the external field, the situation of extraordinary optical transmission in optical wavelength can be investigated.

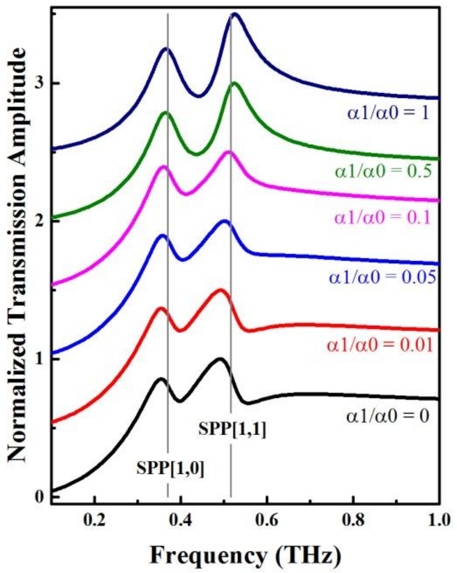

Fig. S2. Normalized modeled transmission spectra varying with increasing α_1, α_2 with fixed α_0 = 0.2, $g_1 = g_2$ = 0.015 THz^2, $\gamma_1 = \gamma_2$ = 0.07 THz, $\omega_0/2\pi$ = 0.43 THz and γ_0 = 0.33 THz. For clarification, the normalized transmission spectra with different α_1, α_2 are vertically shifted by 0.5 respectively. The vertical lines denote the theoretical values of the SPP resonance frequencies of the [1, 0] and [1, 1] modes.

Transmission difference spectra and transmission modulation range

The spectra of measured transmission difference between the periodic and random hole arrays with varying L_y under different illuminations are shown in Fig. S3. Eliminating the impact

from the localized resonance, the transmission enhancement and suppression induced by the coupled SPP modes are obvious, which shows significant Fano feature. As shown in Fig. S3(a), taking the spectrum of L_y = 120 μm as an example, the transmission modulation M_n (n = 1, 2) of each SPP mode can be extracted by measuring the difference between the maximum and minimum values around each SPP mode from the transmission difference spectrum.

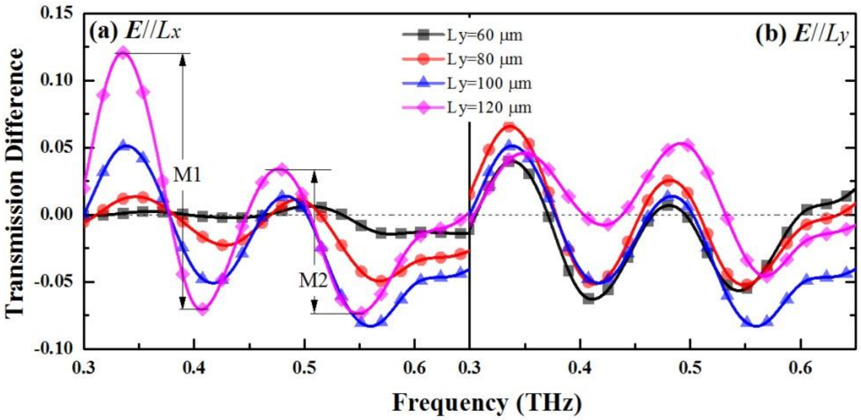

Fig. S3. The spectra of measured transmission difference between the periodic and random hole arrays with varying L_y under ***E***//L_x (a) and ***E***//L_y (b), respectively. Especially, taking the spectrum of L_y = 120 μm as an example, the transmission modulation M_1 and M_2 of the SPP [1, 0] and [1, 1] mode, respectively, are shown in (a).
